# Association between Periodontitis and COVID-19 Based on Severity Scores of HRCT Chest Scans

**DOI:** 10.3390/dj10060106

**Published:** 2022-06-10

**Authors:** Supriya Mishra, Vineeta Gupta, Waheda Rahman, M. P. Gazala, Sukumaran Anil

**Affiliations:** 1 Department of Periodontics, Government Dental College and Hospital, Raipur 492001, India; dr.supriya4@gmail.com (S.M.); vinleo76@gmail.com (V.G.); wahida.505.wr@gmail.com (W.R.); gazala196@gmail.com (M.P.G.); 2 Department of Dentistry, Oral Health Institute, Hamad Medical Corporation, Doha 3050, Qatar; 3 College of Dental Medicine, Qatar University, Doha 2713, Qatar

**Keywords:** COVID-19, cytokine storm, high-resolution computed tomography, periodontitis, risk predictor

## Abstract

Background: A relationship between periodontitis and COVID-19 may exist, as highlighted by several hypothetical models. However, the evidence is limited. Hence, the present study was conducted to determine whether an association exists between periodontitis and COVID-19. Methods: A cross-sectional study was carried out with patients diagnosed with COVID-19 who were divided into three groups—mild, moderate, and severe COVID-19—based on the COVID-19 severity score of high-resolution computed tomography (HRCT) chest scans. Periodontal parameters—including the plaque index (PI), ratio of sites with gingival bleeding (BOP), pocket depth (PD), gingival recession (REC), clinical attachment loss (CAL), and mean numbers of mobile and missing teeth due to periodontitis—were recorded for all three groups. Statistical analyses were applied to the data. Results: Of 294 patients with COVID-19, approximately 50.68% (*n* = 149) had periodontitis, and the highest percentage (87.5%) was reported in the severe COVID-19 group. Additionally, severe and advanced stages of periodontitis (stage III–IV) were found to be significantly more frequent in subjects with severe COVID-19 than in the other two groups. The HRCT severity score (CT-SS) was moderately correlated with increased levels of periodontal parameters. Conclusions: Results of logistic regression analyses showed that the probability of developing severe COVID-19 was 2.81 times higher in patients with periodontitis. An association exists between periodontitis and severe COVID-19.

## 1. Introduction

Coronavirus disease 2019 (COVID-19), caused by SARS-CoV-2—a health problem that poses formidable challenges worldwide—was first identified in the city of Wuhan, China, in December 2019 [1]. Even though SARS-CoV-2 infection can affect multiple organ systems, the lungs are undoubtedly the most commonly affected organs [2]. While most cases result in mild, flu-like symptoms, some cases progress to severe pneumonia and acute respiratory distress syndrome (ARDS), culminating in the death of the patient [3].

The real-time reverse-transcriptase polymerase chain reaction (rRT-PCR) test is a powerful tool that is used as the standard diagnostic test to confirm COVID-19 disease [4]. Additionally, evidence-based research has demonstrated that the use of non-contrast high-resolution computed tomography (HRCT) chest imaging can play an important role in the early identification of the disease and management of patients affected by COVID-19 [1]. Typically, chest CT findings in COVID-19 patients are bilateral, peripheral, and basal predominant ground-glass opacities with or without consolidation and bronchovascular thickening [5]. Due to its high sensitivity, this method is also ideal for assessing the severity of pneumonia in patients with confirmed COVID-19, as well as for monitoring the course of the disease [1]. The disease burden can be ascertained based on the percentage of affected lung volume, using deep learning algorithms [6,7]. The HRCT severity score (CT-SS) has also exhibited a significant positive correlation with clinical symptoms and laboratory markers such as C-reactive protein, serum ferritin, lymphopenia, and D-dimer values, as well as duration of hospital stay, and oxygen requirement in patients with COVID-19 [8,9,10,11].

In terms of studying pathophysiology, the COVID-19 pandemic has posed a challenge unlike any other [12]. With no solution in sight, identifying and delineating factors that may modify the course of the disease to aid in its subsequent care remains an important area of study [13]. Even with the introduction of COVID-19 vaccines, this might continue to be an area of significant concern [13]. Recent relevant studies have demonstrated the association between the severe clinical course of COVID-19 and chronic diseases such as cardiovascular disease, hypertension, diabetes mellitus, obesity, and chronic renal disease [14]. It is possible that there could also be an association between COVID-19 and periodontitis—a common and chronic oral disease in adults.

According to the 2016 Global Burden of Disease Study, severe periodontal disease is the world’s 11th most prevalent disease. [15]. Despite its nonfatal and chronic nature, the disease process is important not only in terms of its effect on oral health, but also for its contribution to the pathophysiology of a variety of systemic diseases. There is enough evidence in the literature to support a link between periodontal disease and the onset and progression of respiratory diseases, including pneumonia and chronic obstructive respiratory disease (COPD) [16]. These mechanistic associations range from direct aspiration of pathogens into the lungs to more indirect mechanisms whereby periodontopathogens modify mucosal surfaces to make them more amenable to colonization, destroy bacterial salivary pellicles to prevent their subsequent clearance, or modify the respiratory epithelium via cytokines to promote infection. [17].

A correlation between periodontitis and COVID-19 has already been stated in several studies from hypotheses to clinical studies and reviews [18,19,20,21,22]. A cytokine response has long been recognized to play a role in the pathogenesis of periodontitis [23]. The stimulation of a “cytokine storm syndrome”, many of the components of which are similar to the cytokine expression profile of periodontitis, has been found to have negative consequences during the COVID-19 pandemic [23]. Periodontal disease appears to be a predictor of serious COVID-19-related outcomes, according to several hypothetical models [24,25]. The successful treatment of severe and critical COVID-19 is indispensable in reducing complications and mortality. Therefore, it is important to determine whether the presence of periodontitis in patients with COVID-19 can be an additional contributing factor (other than those already known in the literature) to disease severity in clinical practice. Thus, the goal of this study was to assess the periodontal status of patients with different degrees of COVID-19, and to determine whether an association exists between periodontitis and COVID-19 severity. A null hypothesis that could be proposed for this specific aim is that periodontal disease is neither a risk factor nor a complicating factor in SARS-CoV-2 infection.

## 2. Materials and Methods

### 2.1. Study Design

The present cross-sectional study was conducted for five months starting from April 2021 until August 2021 in the Outpatient Department of Periodontics, Government Dental College and Hospital, Raipur, Chhattisgarh, India. Scientific approval was obtained from the institution’s ethical review board before the commencement of the study, and was in line with the revised 2013 Helsinki Declaration (ECB/2334/GDC/CG/12.04.2021).

COVID-19-positive patients registered in the state’s COVID-19 monitoring system by the Department of Health and Family Welfare were recruited for the current study. The authors S.M. and V.G., who are registered doctors in the COVID-19 monitoring system and deliver their services to patients diagnosed with COVID-19, obtained the contact details of the patients. The patients were contacted via telephone; a brief introduction and the objectives of the research were presented to them, and they were asked for their consent to participate in the study. Once informed consent was given by the patients, further screening data for the study were collected. These included the patients’ demographic information (age and sex) and whether rRT-PCR testing for the diagnosis and HRCT chest scans to assess the severity of COVID-19 had been administered. Only those patients confirmed to be positive for COVID-19 by rRT-PCR, who were ≥18 years of age and had undergone HRCT chest imaging within 9–13 days of infection (based on the four chronological disease stages, where the peak lung involvement in COVID-19 occurred 9–13 days from the onset of symptoms), were included for further periodontal examination [2]. It was decided to exclude pregnant females from the study.

The CT-SS was based on a semiquantitative scoring system to assess the area of lobar involvement in each of the five lung lobes [2,26]. The scores were assigned on a scale of 0–5, with 0 denoting no involvement, 1 denoting less than 5% involvement, 2 denoting 5–25% involvement, 3 denoting 26–49% involvement, 4 denoting 50–75% involvement, and 5 denoting >75% involvement. The sum of the individual lobar scores represented the total CT-SS, and ranged from 0 (no involvement) to 25 (maximum involvement). Based on the CT-SS for COVID-19, the subjects were stratified into three groups: group I comprised mild COVID-19 patients with scores of 1–8, group II comprised moderate COVID-19 patients with scores of 9–15, and group III comprised severe COVID-19 patients with scores ≥ 15.

### 2.2. Assessment of Covariates

Certain factors have been associated with the severity of COVID-19 disease in the literature. These variables include demographic factors (age and sex), body mass index (BMI), smoking status, and comorbidity with certain medical conditions, including diabetes mellitus (DM), hypertension (HT), cardiovascular and cerebrovascular disorders, asthma and chronic lung diseases, neurological disorders, neoplasia, gastrointestinal illnesses, and pregnancy. Hence, before the clinical examination, the data regarding these covariates were also obtained. The BMI of each participant was calculated by dividing the subject’s body weight (in kilograms) by their height squared (in meters), and categorized as overweight/obese (BMI 23.0–24.9 kg/m^2^/ ≥ 25 kg/m^2^) and adequate/underweight (BMI 18.5–22.9 kg/m^2^)/ < 18.5 kg/m^2^) as per revised Indian guidelines. Smoking status was recorded as current smoker or former/never smoker. A complete medical history was obtained from each patient regarding the presence or absence of systemic diseases. Apart from DM and HT, other systemic disorders were grouped as “other comorbidities”.

### 2.3. Clinical Examination

Participants were scheduled for periodontal examination only when a negative rRT-PCR result showed that they were disease-free. Periodontal examination was carried out by a single, calibrated author (W.R.) who was blinded to the study design. Calibration was performed beforehand on six randomly selected subjects with periodontitis who were not part of the present study, before the clinical examination of the study’s subjects. Two measurements were obtained for PD and CAL at an interval of 24 h. The calibration was found to be acceptable, as 93% of PD and 91% of CAL measurements were within a difference of 1.00 mm. All permanent teeth except for the third molars were examined for recording clinical parameters—plaque index (PI), sites with gingival bleeding (BOP), probing pocket depth (PD), gingival recession (REC), clinical attachment level (CAL), number of mobile teeth, and number of missing teeth due to periodontitis. A mouth mirror and a graduated periodontal probe (UNC-15 probe, Hu-Friedy Manufacturing Co., Chicago, IL, USA) were used for the intraoral examination. The measurements were rounded to the nearest millimeter. Criteria given by Silness and Loe for plaque scores were determined for the labial, lingual, and interproximal surfaces [27], while BOP was evaluated by the presence or absence of bleeding from the gingival sulcus upon probing for 30 s, and computed as the ratio of sites with gingival bleeding to the sites examined [19]. For PD, REC, and CAL, the measurements were taken at six sites per tooth. A tooth was recorded as missing due to periodontal disease when it was absent, and the subject gave a history of mobility in that particular tooth in the absence of any trauma, or if the tooth was extracted due to the presence of periodontal disease. Full-mouth intraoral periapical radiographs were taken in all patients using the long-cone technique for the identification of alveolar bone loss. The patients’ oral hygiene practices were also determined based on the frequency of daily tooth brushing. The clinical case definitions for periodontitis, gingivitis, and periodontal health were based on the diagnostic criteria provided at the 2017 World Workshop on the Classification of Periodontal and Peri-Implant Diseases and Conditions [28]. All of the subjects with periodontitis were further classified as having stage I, II, III, or IV periodontitis based on the staging criteria suggested by Tonetti et al. [29].

### 2.4. Sample Size Calculation and Statistical Analysis

The MedCalc sample size calculator version 20.011 was used to compute the target sample size. This was based on the results of the study performed by Marouf et al. [18], in which the proportion of subjects with periodontitis who had COVID-19 complications was reported to be 0.13. At a 0.95 confidence level (alpha of 0.05), the minimum sample size required was 174. SPSS was used to conduct statistical analyses (version 22.0, IBM Corp, Armonk, NY, USA). Categorical variables were summarized with counts (n) and percentages (%), while continuous variables were summarized by the mean and standard deviation (SD). The normality of the data was checked using the Shapiro–Wilk test. The chi-squared test was used for the comparison of categorical variables, while one-way analysis of variance (ANOVA) was used for making comparisons of the normally distributed continuous variables between the three COVID-19 groups. Pearson’s correlation coefficient was used to determine the correlation between CT-SS and periodontal parameters. Multinomial logistic regression analysis was performed to identify the degree of association between periodontitis and COVID-19. Factors known to be linked with COVID-19 disease severity, as described earlier, were included in the multivariate model irrespective of their significance in the univariate model. The crude and adjusted odds ratios (ORs) and 95% confidence intervals (CIs) were calculated; *p*-values < 0.05 were considered to be significant.

## 3. Results

Out of 1088 screened COVID-19-positive patients, 121 were less than 18 years of age, 197 were diagnosed as COVID-19-negative by other testing methods, and 254 did not have HRCT chest scan reports; hence, these patients were excluded from the present study, resulting in a sample of 516 patients who satisfied the inclusion criteria. Of those 516 subjects, 312 gave their consent to participate in the study; however, 18 patients succumbed to COVID-19 illness. Therefore, a total of 294 patients underwent clinical examination. Of the 294 participants with complete data, 163 had mild COVID-19, 83 had moderate COVID-19, and 48 had severe COVID-19. Summary data from the three groups are presented in Table 1. The mean age (51.87 ± 7.08 years, *p* < 0.0001) and BMI (22.57 ± 1.23, *p* = 0.001) of the participants with severe COVID-19 were significantly higher than those of the other groups. Additionally, a significantly higher number of diabetic (56.25%, *p* = 0.002) and hypertensive patients (56.25%, *p* = 0.0003) reported being affected by severe COVID-19 than in the other groups. Significantly more patients with mild COVID-19 (41.10%) practiced oral hygiene twice daily (*p* = 0.04). Other variables were found to be insignificant among the three groups.

A comparison of periodontal parameters between the three groups is presented in Table 2. Of the 294 COVID-19-positive participants, 149 (50.68%) were diagnosed with periodontitis, 79 (26.87%) had gingivitis, and 66 (22.45%) exhibited periodontal health. Among the 149 periodontitis patients, the group with severe COVID-19 had a significantly higher number of individuals with periodontitis (87.5%) than the other two groups (*p* < 0.0001). There were 79 patients with gingivitis, and a greater number of subjects with moderate COVID-19 (30%) had gingivitis. This association was weakly significant (*p* = 0.048). While the frequency of patients with generalized stage I–II periodontitis was higher in the mild COVID-19 group (84.44%, *p* < 0.0001), a significantly greater number of patients with severe COVID-19 exhibited generalized stage III–IV periodontitis (81.82%, *p* < 0.0001).

Furthermore, when clinical parameters were compared between the three groups of patients with stage I–II periodontitis, the mean PD and CAL were found to be significantly greater in the severe COVID-19 pneumonia group (PD: 3.74 ± 0.12, *p* < 0.0001; CAL: 3.84 ± 0.31, *p* = 0.003). Similarly, the mean PD, CAL, and mean number of mobile teeth were significantly higher in stage III–IV periodontitis patients with severe COVID-19 pneumonia (PD: 5.22 ± 0.47, *p* = 0.001; CAL: 5.71 ± 0.53, *p* = 0.01; mean number of mobile teeth: 4.63 ± 1.20, *p* = 0.006). Details are presented in Table 3.

Regardless of the stage of the disease, the results also showed a significant moderate positive correlation of CT-SS with increasing periodontal parameters, i.e., when PI increased by 0.57 (*p* < 0.0001), the ratio of sites with BOP increased by 0.53 (*p* < 0.0001), PD increased by 0.63 mm (*p* < 0.0001), REC increased by 0.54 mm (*p* < 0.0001), CAL increased by 0.64 mm (*p* < 0.0001), and there was a one-unit increase in CT-SS (Table 4).

The unadjusted odds ratio (OR) of having severe COVID-19 in periodontitis patients was 9.0935 (3.7276 to 22.1832 at 95% CI, *p* < 0.0001). A multinomial logistic regression model for severe COVID-19 among periodontitis patients, which adjusted for all of the covariates, resulted in an adjusted odds ratio of 2.8133 (0.4077–19.7523 at 95% CI, *p* = 0.004), and only age emerged as a significant predictor of the development of severe COVID-19 disease in patients with periodontitis (Table 5). The overall model fit was adequate, with Nagelkerke R^2^ = 0.4604, *p* < 0.0001. There was no significant correlation between periodontitis and moderate COVID-19 (*p* > 0.05) or periodontitis and mild COVID-19 (*p* > 0.05).

## 4. Discussion

A growing body of evidence indicates that certain individuals are at greater risk of suffering severe outcomes as a result of COVID-19 than others. Certain patient characteristics—including increased age, male sex, obesity, symptoms, vital sign parameters, and the presence of several chronic medical conditions, such as diabetes mellitus, hypertension, and chronic lung disorders—are related to an increased risk of severe COVID-19 and its adverse outcomes [24,30]. Hence, a concerted effort has been made to profile these high-risk individuals by developing various COVID-19 models for diagnosis and prognosis. The current study aims to further the literature by demonstrating that periodontitis is another risk factor for developing COVID-19.

The present study found a significant association between periodontitis and severe COVID-19 infection. Periodontitis was found to be more prevalent and severe in patients with severe COVID-19, implying that periodontitis may have an effect on the course and outcome of COVID-19. After adjusting for the common predictors that could influence COVID-19 severity, the correlations between periodontitis and severe COVID-19 remained significant. However, periodontitis was not associated with either moderate COVID-19 or mild COVID-19.

Only a few studies have been conducted in the past to assess the relationship between periodontitis and COVID-19 and its adverse consequences; the present study is one of those few. Our findings were consistent with earlier reports [13,18,19]. Marouf et al. [18] used only one parameter—interdental bone loss (from archived orthopantomographs)—to determine the periodontal status of the subjects, and this may have resulted in diagnostic inaccuracy. However, the present study involved a comprehensive periodontal examination that included the measurement of the clinical periodontal parameters as well as the determination of alveolar bone loss for classification of patients by stage of periodontitis. In a study conducted by Anand et al. [19], patient classification was performed solely based on the presence or absence of COVID-19, regardless of the severity of the disease, while in the present study, patients with varying degrees of COVID-19 based on HRCT chest imaging and severity scores were compared, providing a better picture of the relationship between periodontitis and COVID-19. To prevent viral transmission, the clinical examination was carried out only after the patient was determined to be negative for COVID-19.

The results of the HRCT severity score were used to determine the severity of COVID-19 in the present study. Pneumonia-like changes are a common finding in COVID-19 patients’ lungs [5,31]. If there is no change in lung CT, then the progression to severe COVID-19 disease is unlikely. The most common finding in lung CT is ground-glass opacity, along with some high-density line-like and flake-like consolidation [7,31]. The bilateral involvement of the lungs, multiple pulmonary lobe involvement, and rapid progression of the lesion are important indicators of the progression to severe disease. Although the majority of clinicians and radiologists unequivocally oppose using CT screening to detect COVID-19, as per the WHO advice, CT chest imaging may be used as part of the diagnostic workup for COVID-19 infection if rRT-PCR testing is unavailable, or if highly suspicious cases of COVID-19 have negative rRT-PCR test results. The gold standard method of rRT-PCR was used in this study to confirm the diagnosis of COVID-19, whereas HRCT chest imaging was used to determine the severity of COVID-19 infection in confirmed COVID-19 patients. HRCT imaging is considered to be an essential tool and the most effective method for detecting lung abnormalities in patients with COVID-19 [31]. Previous studies revealed a high correlation of CT-SS with both clinical and laboratory findings, oxygen requirements, and duration of hospital stay, suggesting its potential role in predicting the outcome of COVID-19 [8,9,10,11].

Studies have listed diabetes mellitus, hypertension, and obesity as the three main systemic conditions identifying COVID-19 patients with the most adverse outcomes, including requiring hospitalization [3,31,32]. Additionally, older age has been recognized as a major risk factor for the development of severe COVID-19 symptoms associated with SARS-CoV-2 infection. [31,33].

The results of the present study also support these findings, as higher numbers of older and obese subjects with diabetes and hypertension were reported to have severe COVID-19. However, multinomial logistic regression analysis demonstrated only age as a significant predictor of severe COVID-19 illness, in addition to periodontitis providing an age-dependent effect. The fact that untreated periodontitis is a chronic low-grade systemic inflammation, and has a definite relationship with these other chronic disorders, is well known; hence, it is not surprising that its presence may have an indirect effect in augmenting the effect of the comorbidities in worsening the COVID-19-related adverse outcomes.

Several previous studies have established the presence of periodontopathic microorganisms in the bronchoalveolar fluid of patients with nosocomial pneumonia, along with the elevated risk of respiratory diseases such as aspiration pneumonia and COPD in subjects with severe periodontitis [34,35,36]. Consequently, several hypothetical models speculate on the direct effect of periodontitis in worsening COVID-19, considering that COVID-19 is an infection caused by a virus that primarily affects the respiratory system [13,21,37].

For any infection to occur, the virus must bind to the host’s cellular receptors. In the case of SARS-CoV-2, this host cell receptor is angiotensin-converting enzyme 2 (ACE2), which is articulated at high levels in the oral cavity (the tongue and gingiva in particular), thereby providing the oral cavity as a possible site of viral transmission [21,38,39]. Moreover, studies have also contemplated the possibility that poor plaque control resulting in the presence of periodontopathic microorganisms in excessive amounts might increase the possibility of mortality in COVID-19 patients, making the assessment of oral health status in such patients a necessity that cannot be overstated [38]. In this study, patients with severe COVID-19 presented with inferior oral hygiene, as indicated by higher plaque scores and greater numbers of sites with gingival bleeding. Aspiration of these periodontal pathogens can potentially facilitate the aggravation of SARS-CoV-2 viral infection via enhanced expression of ACE2 [18,19,21].

Additionally, certain reports have suggested that periodontal pathogens do not infect the lungs, but that their constant aspiration might result in the excess production of inflammatory cytokines such as IL-6 and IL-8 by respiratory cells [40]. This excess production of cytokines, known as a cytokine storm, has been considered to be the major cause of aggravation of COVID-19, resulting in acute respiratory distress syndrome (ARDS) and increased mortality [13,40]. The proteases of aspirated periodontopathic bacteria can also exacerbate the infectiousness of SARS-CoV-2 by emphasizing the degradation of the S protein of the virus [21]. Hence, it is suggested that COVID-19-positive patients should initiate vigorous oral hygiene maintenance programs, as this might help reduce the worsening of COVID-19 outcomes. In the present study, a greater number of patients who practiced oral hygiene measures twice daily exhibited mild COVID-19.

One major drawback of this study was that the patients were not evaluated for periodontal status during the active phase of the viral disease. However, since periodontitis is a chronic inflammatory illness, it is presumed the disease would have been present in individuals even before they contracted COVID-19. Additionally alveolar bone loss was not taken into consideration in this study. Second, the study could not obtain data for individuals who developed periodontitis during their illness. We examined a relatively small number of patients with severe COVID-19, which could have caused the results to be underestimated. Additionally, the link between periodontitis and COVID-19 does not imply causation, but it does suggest that more research is needed to better understand the nature and causes of the disease. This could be useful in terms of the early detection and management of problems in COVID-19 patients, as well as in clinical decision making.

## 5. Conclusions

According to the findings of this study, it can be concluded that periodontitis is associated with severe COVID-19, and determining whether the effect is direct or indirect still requires further research. Nevertheless, the evaluation and appropriate management of individuals’ periodontal status should be a part of the COVID-19 treatment protocol, as periodontitis could be a risk factor for severe COVID-19, strengthening the need for maintaining oral health and the commitment to seek periodontal care.

## 6. Patents

**Footnotes**: ¶-UNC-15 probe, Hu-Friedy Manufacturing Co., Chicago, IL, USA.

## Figures and Tables

**Table 1:** Participant details among the three COVID-19 pneumonia groups.

		COVID-19 Pneumonia			
Variable		Mild	Moderate	Severe	*p*-Value
		(*n* = 163)	(*n* = 83)	(*n* = 48)	
Age (in years)		38.41 ± 7.31	41.42 ± 7.72	51.87 ± 7.08	<0.0001 **
Gender (*n*/%)					
	Male	84 (51.53%)	43 (51.8%)	27 (56.25%)	
	Female	79 (48.47%)	40 (48.19%)	21 (43.75%)	0.84
BMI (Kg/m^2^)		21.86 ± 1.09	22.18 ± 1.33	22.57 ± 1.23	0.001 *
Smoking status (*n*/%)					
	Yes	39 (23.93%)	20(25.10%)	15 (31.25%)	
	No	124 (76.07%)	63 (75.90%)	33 (68.75%)	0.57
Diabetes status (*n*/%)					
	Yes	47 (28.83%)	32 (38.55%)	27 (56.25%)	
	No	116 (71.17%)	51 (61.45%)	21 (43.75%)	0.002 *
Hypertension (n/%)					
	Yes	44 (27%)	22 (26.50%)	27 (56.25%)	
	No	119 (73%)	61(73.50%)	21 (43.75%)	0.0003 *
Other co-morbidities					0.59
	Yes	14 (8.58%)	10 (12.05%)	6 (12.5%)	
	No	149 (91.41%)	73 (87.95%)	42 (87.5%)	
Oral hygiene practice					
	Once daily	96(58.89%)	62 (74.69%)	33 (68.75%)	
	Twice daily	67(41.10%)	21 (25.31%)	15 (31.25%)	0.04 *

*n* = counts, %: percentage, mod. = moderate, SD: standard deviation, kg: kilogram, m: meter. *: Fisher’s exact test used, *p* < 0.05 is considered significant; **: one-way ANOVA used, *p* < 0.05 is considered significant.

**Table 2:** Comparison of periodontal parameters between the three COVID-19 pneumonia groups.

	COVID-19 Pneumonia			
Variable	Mild	Moderate	Severe	*p*-Value
	(*n* = 163)	(*n* = 83)	(*n* = 48)	
Periodontitis (149)	70 (42.94%)	37 (44.58%)	42 (87.5%)	<0.0001 *
Gingivitis (79)	48 (29.44%)	25 (30.12%)	06 (12.5%)	0.048 *
Healthy (66)	45 (27.60%)	21 (25.30%)	0 (00.00%)	0.598
Frequency				
Stage I–II (n/%)	45 (64.28%)	15 (40.54%)	09 (21.43%)	<0.0001 *
Stage III–IV (n/%)	25 (35.72%)	22 (59.46%)	33 (78.57%)	
Frequency				
Stage I–II (n/%)				
Localized	07 (15.56%)	04 (26.67%)	03 (33.33%)	0.37
Generalized	38 (84.44%)	11 (73.33%)	06 (66.67%)	
Stage III–IV (n/%)				
Localized	06 (24%)	05 (22.72%)	06 (18.18%)	0.84
Generalized	19 (76%)	17 (77.28%)	27 (81.82%)	
Mean PI				
Healthy	0.25 ± 0.20	0.37 ± 0.33	X	0.18
Gingivitis	1.44 ± 0.64	1.85 ± 0.35	1.92 ± 0.40	<0.0001 *
Periodontitis	1.90 ± 0.35	2.02 ± 0.43	2.11 ± 0.45	0.002 *
Mean BOP				
Healthy	0.03 ± 0.06	0.10 ± 0.12	X	0.055
Gingivitis	0.60 ± 0.30	0.78 ± 0.18	0.90 ± 0.13	<0.0001 *
Periodontitis	0.80 ± 0.25	0.86 ± 0.26	0.90 ± 0.16	0.02 *
Mean PD				
Healthy	1.91 ± 0.36	2.12 ± 0.45	X	0.42
Gingivitis	2.40 ± 0.24	2.5 ± 0.16	3.0 ± 0.29	<0.0001 *
Periodontitis	3.59 ± 0.89	4.48 ± 0.80	4.9 ± 0.75	<0.0001 *
Mean REC				
Healthy	0.03 ± 0.07	0.11 ± 0.25	X	0.27
Gingivitis	0.01 ± 0.03	0.04 ± 0.11	0	0.18
Periodontitis	0.73 ± 0.71	0.87 ± 0.69	1.11 ± 0.78	0.00 *
Mean CAL				
Healthy	0.02 ± 0.05	0.05 ± 0.10	X	0.87
Gingivitis	0.01 ± 0.03	0.05 ± 0.14	0	0.14
Periodontitis	3.75 ± 1.19	4.73 ± 1.07	5.31 ± 0.93	<0.0001 *

*n* = counts, %: percentage, PI: plaque index, BOP: sites with bleeding on probing, PD: pocket depth, REC: recession, CAL: clinical attachment loss, X: nil, *: *p*-values < 0.05 are considered to be significant. The mean values of all periodontal parameters—PI, ratio of sites with BOP, PD, REC, and CAL—were significantly higher in the severe COVID-19 group (PI: 2.11 ± 0.45, *p* = 0.002; BOP: 0.90 ± 0.16, *p* = 0.02; PD: 4.9 ± 0.75, *p* < 0.0001; REC: 1.11 ± 0.78, *p* = 0.005; CAL: 5.31 ± 0.93, *p* < 0.0001). Comparing the periodontal parameters for gingivitis patients between the three groups, the mean PI scores and the ratio of sites with BOP and PD were significantly higher among patients in the severe COVID-19 group than among the patients belonging to the other two groups (PI: 1.92 ± 0.40, *p* < 0.0001; sites with BOP: 0.90 ± 0.13, *p* < 0.0001; PD: 3.0 ± 0.29, *p* < 0.0001).

**Table 3:** Comparison of periodontal parameters of periodontitis patients between the three COVID-19 groups.

Variable	COVID-19 Pneumonia			*p*-Value
	Mild	Moderate	Severe	
Stage I–II Periodontitis				
Mean PI	1.82 ± 0.33	1.83 ± 0.47	1.92 ± 0.22	0.74
Mean BOP	0.72 ± 0.15	0.73 ± 0.18	0.74 ± 0.22	0.94
Mean PD	3.08 ± 0.53	3.61 ± 0.29	3.74 ± 0.12	<0.0001 *
Mean REC	0.27 ± 0.34	0.27 ± 0.35	0.40 ± 0.35	0.81
Mean CAL	3.03 ± 0.75	3.69 ± 0.49	3.84 ± 0.31	0.003 *
Mobile teeth	0.68 ± 0.87	1.20 ± 0.97	1.0 ± 1.00	0.15
Missing teeth	0.44 ± 0.69	0.53 ± 0.91	0.33 ± 0.57	0.88
Stage III–IV Periodontitis				
Mean PI	2.05 ± 0.35	2.15 ± 0.36	2.16 ± 0.49	0.59
Mean BOP	0.79 ± 0.16	0.83 ± 0.16	0.86 ± 0.14	0.43
Mean PD	4.55 ± 0.66	5.07 ± 0.37	5.22 ± 0.47	0.001 *
Mean REC	1.11 ± 0.82	1.27 ± 0.76	1.30 ± 0.76	0.71
Mean CAL	5.04 ± 0.57	5.44 ± 0.72	5.71 ± 0.53	0.01 *
Mobile teeth	2.76 ± 1.66	4.04 ± 1.96	4.63 ± 1.20	0.006 *
Missing teeth	1.20 ± 1.35	1.63 ± 1.78	2.27 ± 2.05	0.21

PI: plaque index, BOP: sites with bleeding on probing, PD: pocket depth, REC: recession, CAL: clinical attachment loss, *: *p*-values < 0.05 are considered to be significant.

**Table 4:** Correlation of HRCT scores with periodontal parameters.

Variable	PI	BOP	PD	REC	CAL
	r (*p*-value)	r (*p*-value)	r (*p*-value)	r (*p*-value)	r (*p*-value)
CT-SS *	0.57	0.53	0.63	0.54	0.64
	(<0.0001)	(<0.0001)	(<0.0001)	(<0.0001)	(<0.0001)

PI: plaque index, BOP: sites with bleeding on probing, PD: pocket depth, REC: recession, CAL: clinical attachment loss, HRCT-SS: high-resolution computed tomography severity score, * *p*-values < 0.05 are considered to be significant.

**Table 5:** Multinomial logistic regression analysis.

Variable	Odds Ratio	95% CI	*p*-Value
Periodontitis	2.81	0.40 to 19.75	0.004 *
Age	1.38	1.20 to 1.58	<0.0001 *
Female gender	1.02	0.21 to 5.10	0.9773
BMI	1.00	0.51 to 1.95	0.9968
Smoking status	5.21	0.87 to 31.32	0.0714
DM status	0.24	0.06 to 1.04	0.0559
HT status	0.46	0.11 to 1.95	0.2955
OHP	1.49	0.37 to 5.9428	0.5702

BMI: body mass index, DM: diabetes mellitus, HT: hypertension, OHP: oral hygiene practice *: *p*-values < 0.05 are considered to be significant.

## Data Availability

The datasets used and/or analyzed during the present study are available from the corresponding author upon reasonable request.

## Abbreviations

COVID-19	Coronavirus disease 2019
ARDS	Acute respiratory distress syndrome
COPD	Chronic obstructive respiratory disease
rRT-PCR	Real-time reverse-transcription polymerase chain reaction
CT-SS	High-resolution computed tomography severity score
HRCT	High-resolution computed tomography
BMI	Body mass index
DM	Diabetes mellitus
HT	Hypertension
BOP	Bleeding on probing
PD	Probing pocket depth
REC	Gingival recession
CAL	Clinical attachment level
ACE2	Angiotensin-converting enzyme 2
